# A Scaling Law for SPAD Pixel Miniaturization

**DOI:** 10.3390/s21103447

**Published:** 2021-05-15

**Authors:** Kazuhiro Morimoto, Edoardo Charbon

**Affiliations:** 1 AQUA Laboratory, École Polytechnique Fédérale de Lausanne (EPFL), 2000 Neuchâtel, Switzerland; 2 Canon Inc., Kanagawa 212-8602, Japan; morimoto.kazuhiro@mail.canon

**Keywords:** single-photon avalanche diode, pixel miniaturization, scaling law

## Abstract

The growing demands on compact and high-definition single-photon avalanche diode (SPAD) arrays have motivated researchers to explore pixel miniaturization techniques to achieve sub-10 μm pixels. The scaling of the SPAD pixel size has an impact on key performance metrics, and it is, thereby, critical to conduct a systematic analysis of the underlying tradeoffs in miniaturized SPADs. On the basis of the general assumptions and constraints for layout geometry, we performed an analytical formulation of the scaling laws for the key metrics, such as the fill factor (FF), photon detection probability (PDP), dark count rate (DCR), correlated noise, and power consumption. Numerical calculations for various parameter sets indicated that some of the metrics, such as the DCR and power consumption, were improved by pixel miniaturization, whereas other metrics, such as the FF and PDP, were degraded. Comparison of the theoretically estimated scaling trends with previously published experimental results suggests that the scaling law analysis is in good agreement with practical SPAD devices. Our scaling law analysis could provide a useful tool to conduct a detailed performance comparison between various process, device, and layout configurations, which is essential for pushing the limit of SPAD pixel miniaturization toward sub-2 μm-pitch SPADs.

## 1. Introduction

Single-photon avalanche diodes (SPADs) have been widely recognized for having unique features, such as single-photon sensitivity and picosecond timing resolution. In recent decades, SPAD arrays fabricated with the silicon-based complementary-metal-oxide-semiconductor (CMOS) process have been extensively studied for a number of scientific and industrial applications. To explore the emerging applications of SPAD image sensors, researchers have developed large-scale SPAD pixel arrays in compact sensor formats. Continuous efforts in SPAD research and development have led to the exponential growth of the array size and dramatic shrinkage of the pixel dimension; the SPAD array size has reached a milestone of 1 megapixel [1], while a SPAD pixel with 2.2 μm-pitch was reported in test devices [2].

In addition, 3D-stacking approaches have enabled the physically isolation of pixel circuits from the SPAD array while ensuring electrical connection via pixel-level bonding, which provides a promising solution for pixel miniaturization below 10 μm [3,4,5,6]. Such an aggressive miniaturization and scaling of SPAD pixels could have a major influence on the key performance of SPADs, and it is, thereby, critical for designers to understand the fundamental tradeoffs in miniaturized SPADs. Theoretical studies on SPAD performance have been widely performed based on both analytical methods and simulations to describe process, voltage, and temperature dependence [7,8,9]. However, few attempts have been made to systematically analyze the impact of SPAD pixel scaling on major performance metrics, such as the fill factor (FF), photon detection probability (PDP), photon detection efficiency (PDE), dark count rate (DCR), correlated noise, and power consumption.

In this paper, we present an in-depth study of scaling laws in SPADs to clarify the underlying tradeoffs in SPAD design and to give perspectives for the future design of multi-megapixel SPAD arrays. The formulation of scaling laws with regard to the pixel size is performed based on the assumption that the pixel circuit can be located outside of the SPAD array and does not impact the pixel layout. Based on the introduced equations, the scaling behavior of the SPAD performance is exemplified in the plots. The scaling law equations are then compared with previously published experimental results with various SPAD sizes. A good agreement between the theoretical fitting and experimental data validates the scaling law analysis.

The paper comprises four sections. Section 2 presents the theoretical formulation of scaling laws for various performance metrics in the SPAD pixels. Some examples of analyzing experimental data with the theoretical expressions is demonstrated in Section 3, and this is followed by discussion in Section 4.

## 2. Scaling Law Analysis

### 2.1. Analysis Criteria

To proceed with the theoretical analysis of the scaling laws, some assumptions must be made. First, the SPAD pixel array configuration is assumed to be a square grid, while it is not difficult to generalize the discussion to other configurations, e.g., honeycomb structure [10]. Second, circular-shaped SPADs are assumed to simplify the discussion on the curvature change with scaling. In some prior works, rounded-corner rectangle or square SPADs are also adopted to improve the fill factor [11,12,13]. However, these designs are not always suitable for scaling with the geometric similarity preserved, where the electric field concentration at the corners can induce premature edge breakdown and also change the breakdown voltage. Third, a 3D-stacked configuration with a SPAD-only array in a single plane is assumed.

In a non-3D-stacked FSI or BSI configuration, SPAD and pixel circuits coexist in the same plane. In a given pixel pitch, the SPAD and circuit have to share the limited area, and the circuit complexity can affect the size of the SPAD active area and its performance. The main focus of this analysis is to formulate the scaling laws of SPAD performance, and, hence, the SPAD array without circuit components is desired for more systematic and quantitative analysis. Fourth, the active-to-active distance is assumed to be fixed at a certain dimension, irrespective of the scaling parameter. This is justified by the following discussion.

For analysis of the scaling laws in the SPAD pixel, it is natural to assume that the doping profile along with the *z*-axis for each implantation layer is unchanged, and the breakdown voltage of the p-n junction in the SPAD remains in the same range. This implies that, unlike scaling in MOS transistors, where a lower supply voltage is adopted for the smaller devices, the power supply voltage for the SPAD does not scale as a function of its dimensions. Another premise in SPAD pixel design is that the guard-ring width is sufficiently large to avoid premature edge breakdown. Given the fact that the lateral diffusion length of doped ions cannot readily be controlled, the electrostatic potential distribution around the guard ring is not dependent on the active diameter. The optimum guard-ring width ensuring no edge breakdown in the operating condition is defined by the following equation [14]:

(1) $$\begin{array}{c} V_{B}^{gr}\left(W_{gr}\right)=V_{B}^{p-n}+V_{ex}^{max}, \end{array}$$

where $V_{B}^{gr}\left(W_{gr}\right)$ is the breakdown voltage at the guard ring with the given guard-ring width $W_{gr}$, $V_{B}^{p-n}$ is the breakdown voltage at the vertical p-n junction, and $V_{ex}^{max}$ is the maximum excess bias used in the system. Based on the discussion above, all the terms in the above equation are not dependent on pixel size, and the optimum $W_{gr}$ can be defined regardless of scaling. These considerations impose a constraint in the pixel scaling that the guard-ring width has to be unscaled and fixed at a certain value over all the SPAD pixel dimensions to guarantee stable Geiger-mode operation without unwanted edge breakdown. The optimum $W_{gr}$ should be comparable to the depletion width of the main SPAD p-n junction, and is typically 1 to 2 μm [15]. In addition, the optimum width of an isolation layer, typically formed with deep-well implantation, is determined by a process design rule for the minimum drawing width, and should not be scaled with the pixel dimensions. The pixel pitch $L_{p}$, which will be employed as a scaling parameter in the following discussion, can be expressed as:

(2) $$\begin{array}{c} L_{p}=D_{a}+L_{a-a}=D_{a}+2\cdot W_{gr}+W_{iso}, \end{array}$$

where the well-sharing configuration is assumed, and $D_{a}$ is the active diameter, $L_{a-a}$ is the active-to-active distance, and $W_{iso}$ is the isolation width. In the following discussion, $W_{gr}$ and $W_{iso}$ are both assumed to be 1 μm unless otherwise noted, and $L_{p}$ is assumed to be solely dependent on $D_{a}$.

**Figure 1:**
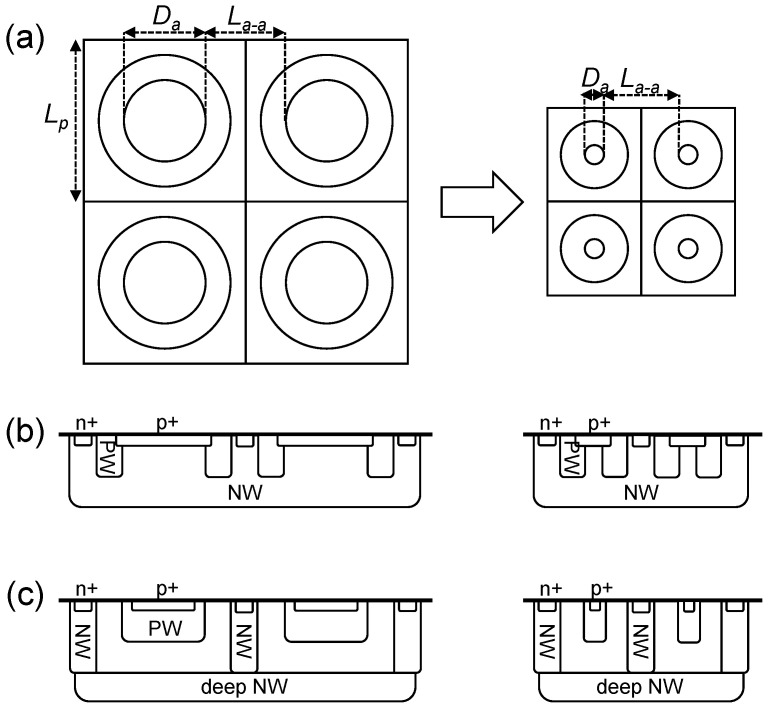
Conceptual views of SPAD pixel scaling; (**a**) top-view layout examples of pixel miniaturization, (**b**) cross-section example of p+/NW SPAD, and (**c**) cross-section example of PW/deep-NW SPAD or p-i-n SPAD.

Figure 1 shows the conceptual views depicting the SPAD pixel scaling. Figure 1a is the example of a top-view layout for a 2 × 2 pixel array. As discussed above, the active-to-active distance $L_{a-a}$ is fixed when shrinking the pixel pitch $L_{p}$. As a result, the active diameter $D_{a}$ is reduced proportionally to $L_{p}$. This assumption can be applied to any type of existing SPAD device structures [16,17]. For example, Figure 1b shows the cross-sectional view of p+/NW SPAD. $D_{a}$ is defined as the diameter of the inner circle of the guard-ring p-well, whereas $L_{a-a}$ corresponds to a sum of the NW separation width and twice the width of the p-well guard ring. For PW/deep-NW SPAD or p-i-n SPAD, $D_{a}$ equals the diameter of the p-well, and $L_{a-a}$ is a sum of the NW separation width and twice the width of the virtual p-epi guard ring. This indicates that the scaling law analysis can be performed with only three key dimensional parameters, $L_{p}$, $D_{a}$, and $L_{a-a}$, without losing generality.

In summary, the main assumptions for the analysis of scaling laws are:

- a uniform square grid,
- a circular shape for the active area and inner/outer borders of the guard ring,
- a 3D-stacked configuration with full separation of the SPAD and pixel circuit into different wafers,
- an active-to-active distance unscaled with the SPAD pixel dimension, and
- the pixel pitch $L_{p}$ employed as a scaling parameter.

### 2.2. Formulation of Scaling Laws

#### 2.2.1. Fill Factor

The FF in the SPAD pixel, defined as the ratio between the drawn active area and the pixel area, is one of the fundamental parameters determining the single-photon sensitivity. FF is a purely geometric parameter, and is straightforward to be formulated as a function of the pixel pitch $L_{p}$:

(3) $$\begin{array}{c} FF=\frac{\pi {\left(L_{p}-L_{a-a}\right)}^{2}}{4L_{p}^{2}}. \end{array}$$

It is clear from the above equation that FF goes down to zero when $L_{p}=L_{a-a}$ and cannot be defined for $L_{p}<L_{a-a}$. For sufficiently large $L_{p}$, FF converges to π/4×100 = 78.5%.

**Figure 2:**
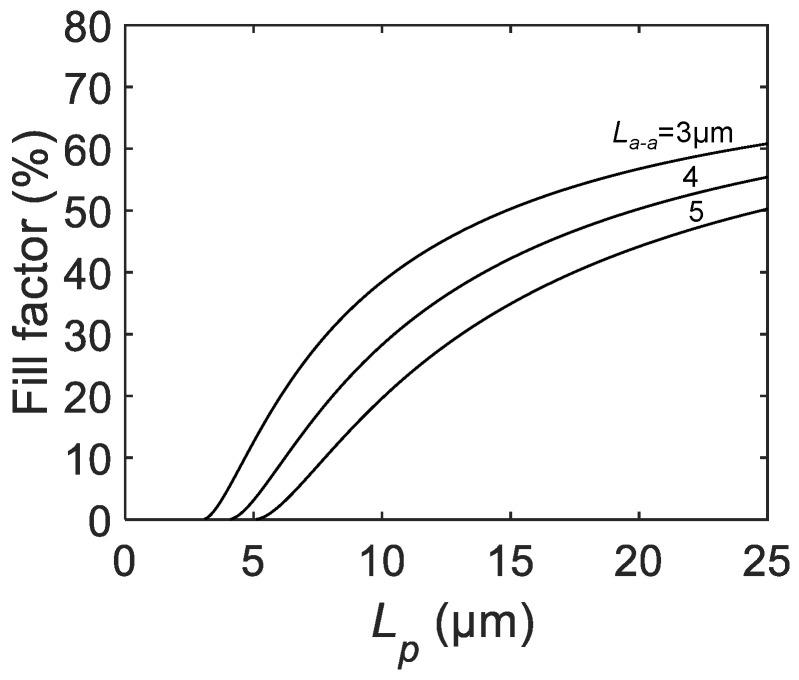
The calculated FF as a function of the SPAD pixel pitch $L_{p}$ for active-to-active distances $L_{a-a}$ = 3, 4, 5 μm.

Figure 2 shows the calculated FF as a function of the pixel pitch for several different active-to-active distances. FF curves show monotonic increases with the pixel pitch $L_{p}$. A relatively steep increase of FF is observed at smaller $L_{p}$, whereas saturating behavior of FF is shown at larger $L_{p}$. Slower saturation for larger $L_{a-a}$ indicates that, if the active-to-active distance is large, a larger pixel pitch is required to obtain a higher FF, e.g., above 50%. In the actual sensor design, the effective FF can be enhanced by employing on-chip microlenses [18,19], although designers should bear in mind that microlenses are less effective for smaller f-numbers of the main objective lens.

#### 2.2.2. PDP and PDE

The PDP in SPAD pixels is defined by the following equation [20]:

(4) $$\begin{array}{c} PDP=QE\times P_{ava}, \end{array}$$

where QE is the quantum efficiency and $P_{ava}$ is the avalanche triggering probability. In ideal SPAD devices, PDP represents the single-photon sensitivity normalized by the active area, and it does not scale with the active diameter and the pixel pitch. In practice, a discrepancy between the “drawn” active area and “effective” active area leads to considerable dependencies of PDP from the scaling parameter $L_{p}$ [21].

The discrepancy between the designed and actual active size stems from two possible reasons: nonideality in the process fabrication and nonideality in the device design. One example of the process nonideality is the lateral diffusion of doped ions [22]. The lateral diffusion length is determined by the type of dopant ions, implantation energy, and thermal annealing conditions and is typically in the order of 0.1 to 1 μm for deep well implantation. This lateral diffusion induces the decrease of the doping concentration at the edge of the active area. The electric field at the edge of the active area can be locally reduced with respect to the electric field at the center of the active area, thus, lowering the sensitivity at the border of the active area.

On the other hand, the device design nonideality is caused by a lateral electric field near the guard ring. Photocharges generated in the neutral region of the SPAD randomly move around due to thermal diffusion until they reach the nearby depletion region and are drifted to an electrode. If the photocharges reach the main p-n junction with a high electric field, they induce avalanche multiplication, thereby generating a photon detection signal. However, photocharges close to the border of the active area can reach the depletion region toward the guard ring before reaching the main junction. In such a case, the carriers do not cause avalanche multiplication, and no photon detection signal is observed. This so-called “border effect” [23,24] causes the photon detection loss at the edge of active area, which becomes more significant in the smaller pixels.

For both process- and device-originated nonidealities, PDP correction can be performed by introducing an inactive radius $r_{in}$, representing the effective width of the photon-insensitive region at the edge of the active region [25]. The corrected equation for the scaling law of PDP is given by:

(5) $$\begin{array}{c} PDP=PDP_{max}\times {\left(\frac{L_{p}-L_{a-a}-2r_{in}}{L_{p}-L_{a-a}}\right)}^{2}, \end{array}$$

where $PDP_{max}$ is the virtual maximum PDP with a sufficiently large active size.

**Figure 3:**
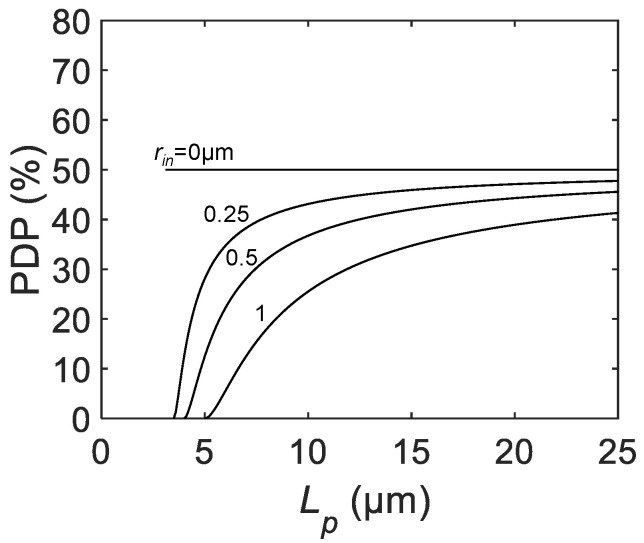
The calculated PDP as a function of the SPAD pixel pitch $L_{p}$ for $PDP_{max}=$ 50%, active-to-active distance $L_{a-a}=$ 3 μm, and inactive radius $r_{in}$ = 0, 0.25, 0.5, and 1 μm.

Figure 3 shows the calculated PDP as a function of the pixel pitch for different $r_{in}$. The curve with $r_{in}=$ 0 μm corresponding to the ideal case with no border effect shows no dependency with $L_{p}$. For finite $r_{in}$, PDP starts from zero at $L_{p}=L_{a-a}+2r_{in}$ and grows and saturates to $PDP_{max}$ with increasing $L_{p}$. Similar to the scaling law for the FF, a slower increase is observed for the larger $r_{in}$.

PDE is another indicator of single-photon sensitivity. Unlike PDP, where the sensitivity is normalized by the active area, PDE is defined as the single-photon sensitivity normalized to the pixel area. The following equation holds [20]:

(6) $$\begin{array}{c} PDE=PDP\times FF. \end{array}$$

Based on the previous equations, PDE can be explicitly formulated as:

(7) $$\begin{array}{c} PDE=PDP_{max}\times \frac{\pi {\left(L_{p}-L_{a-a}-2r_{in}\right)}^{2}}{4L_{p}^{2}}. \end{array}$$

**Figure 4:**
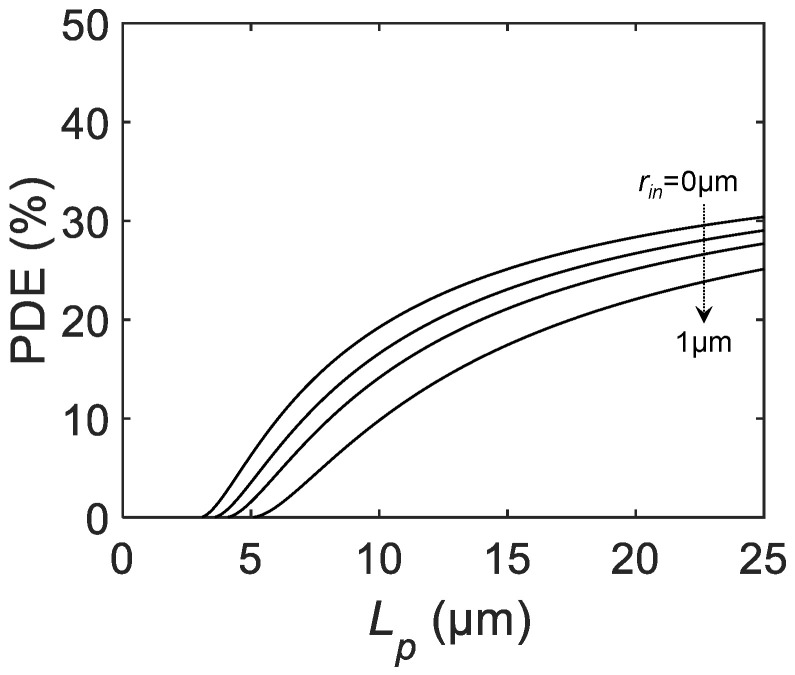
The calculated PDE as a function of the SPAD pixel pitch $L_{p}$ for $PDP_{max}=$ 50%, active-to-active distance $L_{a-a}=$ 3 μm, and inactive radius $r_{in}$ = 0, 0.25, 0.5, and 1 μm.

Figure 4 is the calculated PDE as a function of $L_{p}$ for different $r_{in}$. Similar to FF and PDP, the curves start from zero at smaller $L_{p}$ and saturate at larger $L_{p}$. The maximum PDE is given by $PDP_{max}\times 78.5\%=39.3\%$, assuming $PDP_{max}=50\%$. Again, introducing on-chip microlenses will potentially increase the overall PDE.

#### 2.2.3. DCR

DCR has several different causes, such as band-to-band tunneling, trap-assisted tunneling, trap-assisted thermal generation, and the diffusion current [26,27]. Experimentally, the source of the DCR can be classified based on an Arrhenius plot [28,29,30]. In silicon SPADs, the activation energies $E_{a}$ for band-to-band tunneling, trap-assisted tunneling, trap-assisted thermal generation, and the diffusion current are known to be approximately 0, 0–0.55, 0.55, and 1.1 eV, respectively. In practice, the measured $E_{a}$ can have intermediate values, e.g., 0.8 eV, indicating a mixture of multiple DCR components.

Based on the assumption that premature edge breakdown is suppressed, the tunneling components at the edge of the active region can be neglected. Contributions of the thermal generation and diffusion current are also negligible in the depletion region to the guard ring due to an insufficient electric field for avalanche triggering by the generated carriers. Therefore, the contribution from the main p-n junction of the SPAD dominates over that from the edge of the active region. Interestingly, all the aforementioned DCR components are proportional to the “effective” active area.

The tunneling current, regardless of being band-to-band or trap-assisted, is proportional to the total volume of the region with a highly concentrated electric field, which is clearly proportional to the active area. Thermal generation and diffusion carriers are detected only when those carriers are generated in the vicinity of the active region. Assuming that thermal generation and the diffusion current are spatially uniform around the active region, those components are also naturally assumed to be proportional to the active area. The scaling law for DCR can be formulated as follows:

(8) $$\begin{array}{c} DCR=R_{0}\times \frac{\pi {\left(L_{p}-L_{a-a}-2r_{in}\right)}^{2}}{4}, \end{array}$$

where $R_{0}$ is the DCR per unit of active area.

**Figure 5:**
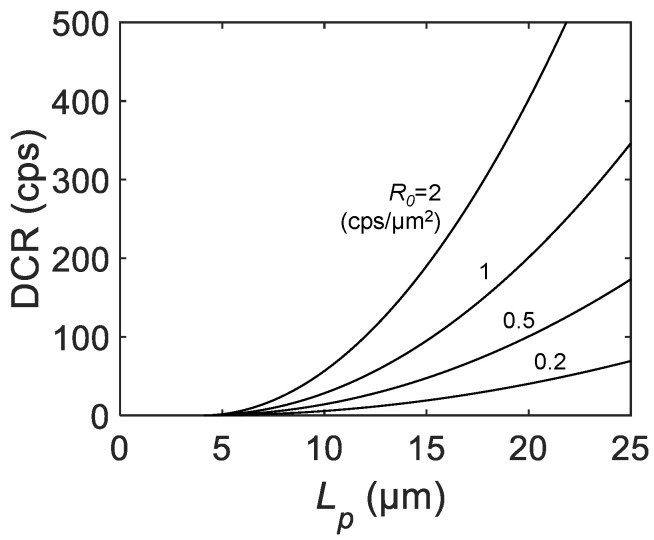
The calculated DCR as a function of the SPAD pixel pitch $L_{p}$ for $R_{0}=$ 0.2, 0.5, 1, and 2 cps/μm${}^{2}$, active-to-active distance $L_{a-a}=$ 3 μm and inactive radius $r_{in}=$ 0.5 μm.

Figure 5 is the calculated DCR as a function of $L_{p}$ for different DCRs per unit area $R_{0}$. Starting from 0 cps at $L_{p}=L_{a-a}+2r_{in}$, the DCR shows a parabolic increase with $L_{p}$. the DCR is highly dependent on $R_{0}$, which is a function of the excess bias, temperature, and process quality, such as the trap and impurity densities. Opposite to the FF, PDP, and PDE, a smaller pixel pitch is desirable to improve DCR performance. The designer should consider the best tradeoff between PDE and DCR to find the optimum $L_{p}$ to, thus, provide a reasonable S/N ratio.

The DCR density *R* is defined by the DCR normalized by the drawn active area and is often used for comparison of the SPAD process quality between devices fabricated in different processes [31]. As with PDP, nonideality, such as for the border effect, leads to the dependence of *R* on $L_{p}$ as follows:

(9) $$\begin{array}{c} R=R_{0}\times \frac{\pi {\left(L_{p}-L_{a-a}-2r_{in}\right)}^{2}}{4{\left(L_{p}-L_{a-a}\right)}^{2}}. \end{array}$$

**Figure 6:**
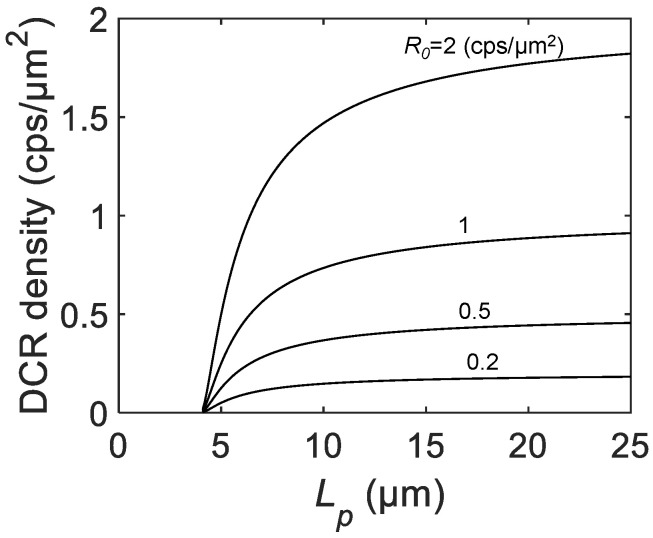
The calculated DCR density as a function of the SPAD pixel pitch $L_{p}$ for $R_{0}=$ 0.2, 0.5, 1, and 2 cps/μm${}^{2}$, active-to-active distance $L_{a-a}=$ 3 μm, and inactive radius $r_{in}=$ 0.5 μm.

At larger $L_{p}$, the DCR density saturates to $R_{0}$. Figure 6 shows the $L_{p}$ dependence of the DCR density for various $R_{0}$. As can be seen from the similarity to the equation for PDP, the DCR density starts from zero at $L_{p}=L_{a-a}+2r_{in}$ and rapidly increases and saturates for larger $L_{p}$. This implies that, in the actual measurement, the DCR density can be underestimated at the smaller pixel pitch due to the existence of the photon-insensitive region at the edge of the active region.

Note that the above discussion is based on the assumption that the guard-ring width is optimized to avoid edge breakdown for the entire range of the pixel pitch. In the actual device design, sometimes an abrupt increase of DCR and DCR density is observed at a smaller pixel pitch even with fixed active-to-active distance. To the best of our knowledge, no systematic analysis has been conducted for this phenomenon. One possible reason is the enhanced curvature at the edge of the active region inducing a high electric field near the guard ring. Analogously to antennas, the electric field tends to increase in regions of high curvature, which may induce premature edge breakdown when scaling down the pixel. Another possible explanation is the nonideality in the photoresist formation process.

In most SPAD devices, the diffusion regions for the p-n junction, guard ring, or isolation are formed by well doping where high energy doping is employed. In such a process, a thicker photoresist is desired to avoid penetration of the accelerated ions through the resist. The opening size of the photoresist for such a thick resist (typically 3 to 10 μm) requires careful calibration to match the actual shape and size to the designed layout. The layout for well doping is usually supported only for 0 or 90 degree lines, whereas a SPAD layout often involves a circular or ring shape with arbitrary angles. This could cause the deviation of the actual resist opening size from the design especially in the smaller pixel dimension, leading to unwanted edge breakdown.

#### 2.2.4. Afterpulsing Probability

Correlated noise, such as afterpulsing and crosstalk, is critical for certain applications where the temporal and spatial correlations of photon detection signals play key roles [32,33,34]. Afterpulsing is caused by an avalanche-generated carrier captured at a deep trap state near the multiplication region, which is released by thermal activation or tunneling after a nanosecond to microsecond trapping time, thus, inducing another avalanche multiplication event. This mechanism implies that the afterpulsing probability $P_{a}$ is dependent on the trap density $D_{trap}$ and the total number of avalanche-generated carriers $N_{ava}$. A higher trap density and more avalanche carriers result in a higher $P_{a}$. If $P_{a}$ is not overly large, e.g., smaller than 10%, a linear relation between $P_{a}$ and $D_{trap}\times N_{ava}$ can be assumed to a first-order approximation [35].

Assuming the spatially uniform distribution of the deep trap states, $D_{trap}$ is independent of the scaling parameter. $N_{ava}$, on the other hand, can be dependent on the scaling parameter. $N_{ava}$ is calculated based on the following:

(10) $$\begin{array}{c} eN_{ava}=C_{par}\times V_{ex}=\left(C_{p-n}+C_{0}\right)\times V_{ex}, \end{array}$$

where *e* is the elementary charge; $V_{ex}$ is the excess bias; $C_{par}$ is the total parasitic capacitance at the SPAD output node, either cathode or anode, which is connected to the quenching resistor; $C_{p-n}$ is the p-n junction capacitance at the active region; and $C_{0}$ is the sum of the other parasitic capacitance contributions from connected metal wires, diffusion regions, gates, etc. $C_{p-n}$ is proportional to the active area, whereas $C_{0}$ does not scale with the pixel size or the active size. In summary, the scaling law of $P_{a}$ is given by:

(11) $$\begin{array}{c} P_{a}=A\times \left[\frac{\pi \epsilon {\left(L_{p}-L_{a-a}\right)}^{2}}{4W_{eff}}+C_{0}\right], \end{array}$$

where *A* is the temperature-, bias-, and process-dependent coefficient; ϵ is the permittivity; and $W_{eff}$ is the effective depletion region width determined by the p-n junction doping profile.

**Figure 7:**
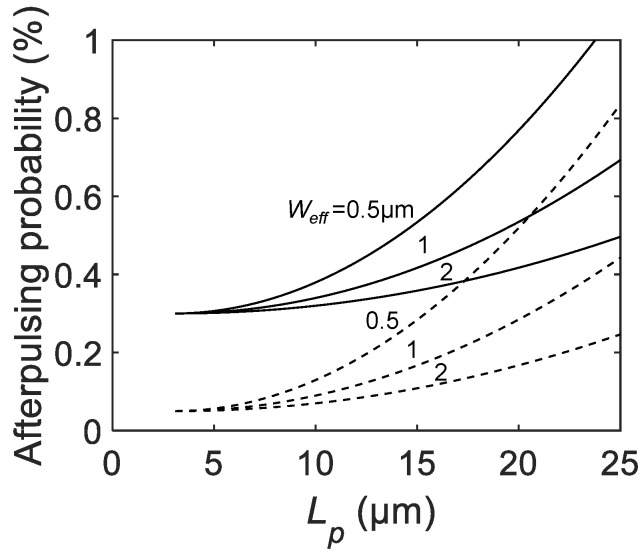
The calculated afterpulsing probability as a function of the SPAD pixel pitch $L_{p}$ for $C_{0}=$ 5 fF (dashed lines), and 30 fF (solid lines), active-to-active distance $L_{a-a}=$ 3 μm, $A=1\times 10^{11}$ F${}^{-1}$, and $W_{eff}=$ 0.5, 1, 2 μm.

Figure 7 shows the $L_{p}$ dependence of the afterpulsing probability for various $W_{eff}$ and $C_{0}$ (dashed lines for $C_{0}=$ 5 fF, and solid lines for $C_{0}=$ 30 fF). For all parameter combinations, the parabolic increase of $P_{a}$ is shown with the offset corresponding to $A\times C_{0}$. A larger $W_{eff}$ shows a weaker dependence of $P_{a}$ on $L_{p}$, indicating less contribution of the p-n junction capacitance to the total parasitic capacitance $C_{par}$. In any case, scaling down of the pixel has a positive impact on the afterpulsing probability due to the reduced parasitic capacitance.

Note that the dead time is assumed to be constant for all $L_{p}$ in this analysis. In a real device design, fixed quenching resistance results in the $L_{p}$ dependence of the dead time. This secondary effect makes the $P_{a}$ less sensitive to $L_{p}$ compared to the case where constant dead time is assumed. If the dependence of $P_{a}$ on the dead time is strong enough to compensate for the trend as shown in Figure 7 then it will be possible to flatten or even reverse $P_{a}$ for larger $L_{p}$.

#### 2.2.5. Crosstalk Probability

Crosstalk is another type of correlated noise in SPAD pixels. Unlike afterpulsing, where only a single pixel is involved, crosstalk involves two or more pixels. When avalanche multiplication is triggered in a pixel, thousands to millions of electrons and holes are generated. When those carriers are recombined with counterpart charges, either photons or phonons can be emitted to preserve the energy conservation law. Silicon is a material with an indirect bandgap, and hence the probability to emit photons is very low. For photon energy higher than the silicon bandgap, only several to tens of photons are emitted out of the one million avalanche-generated carriers [36,37]. However, those photons can move toward a neighboring pixel and be detected.

Similar to afterpulsing, the crosstalk probability $P_{c}$ is dependent on the number of avalanche-generated carriers $N_{ava}$. A larger number of carriers leads to a higher $P_{c}$. Again, to a first-order approximation, $P_{c}$ is considered to be proportional to $N_{ava}$. In addition, the distance between pixels is another important factor for scaling. Given that the emitted secondary photons decay exponentially with the travel length, a shorter pixel-to-pixel distance could result in higher crosstalk. The emitter-to-receiver distance dependence of crosstalk can be approximated by [38]:

(12) $$\begin{array}{c} P_{c}=B\frac{e^{-\alpha r}}{r^{2}}, \end{array}$$

where *B* is a coefficient that will be explained later, *r* is the distance from one SPAD of interest to the other, and α is the effective decay length of the emitted light. Regarding the crosstalk between two nearest-neighbor SPAD pixels, *r* in the above equation corresponds to the pixel pitch $L_{p}$. Note that this equation implicitly assumes that the light emission occurs at the center of the active region for the emitting SPAD, and the average photon intensity reaching the active region of receiver is approximated by the photon intensity at the center of the active region of the receiving SPAD. In reality, the finite size of the active region for both the emitter and receiver may cause a slight deviation of the measured crosstalk from the above model. For simplicity, the following analysis will be based on the above model where the effect of the finite active size is neglected.

The coefficient *B* is dependent on both the emitter and receiver characteristics. Considering the emitter, *B* should depend on the total number of emitted photons, which is proportional to $N_{ava}$. On the other hand, *B* should also be correlated with the sensitivity of the receiver. The probability of detecting an emitted photon is proportional to the PDP and the active area, which coincide with the PDE times $L_{p}^{2}$ by definition. Thus, the crosstalk probability between two nearest-neighbor SPAD pixels can be expressed as:

(13) $$\begin{array}{c} P_{c}=B^{\prime }\times \left[\frac{\pi \epsilon {\left(L_{p}-L_{a-a}\right)}^{2}}{4W_{eff}}+C_{0}\right]\times \frac{e^{-\alpha L_{p}}}{L_{p}^{2}}\times \frac{\pi }{4}\times {\left(L_{p}-L_{a-a}-2r_{in}\right)}^{2}, \end{array}$$

where $B^{\prime }$ is an excess-bias dependent coefficient.

**Figure 8:**
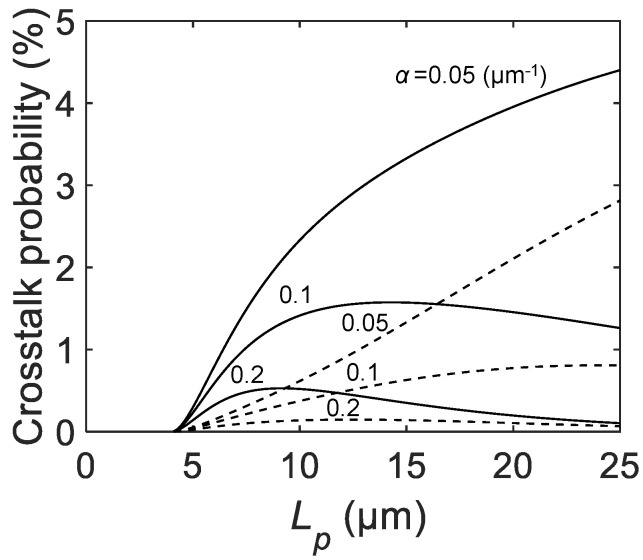
The calculated crosstalk probability as a function of the SPAD pixel pitch $L_{p}$ for $C_{0}=$ 5 fF (dashed lines) and 30 fF (solid lines), active-to-active distance $L_{a-a}=$ 3 μm, $B^{\prime }=4\times 10^{12}$ F${}^{-1}$, $W_{eff}=$ 1 μm, $r_{in}$ = 0.5 μm, and α= 0.05, 0.1, and 0.2 μm${}^{-1}$.

Figure 8 shows the $L_{p}$ dependence of the calculated crosstalk probability for various α and $C_{0}$. All curves show increasing trends for $L_{p}$ close to $L_{a-a}+2r_{in}$. For larger $L_{p}$, either increasing or decreasing trends are observed, depending on the parameter set. The curve with α= 0.2, 0.1 μm${}^{-1}$, and $C_{0}=30$ fF shows reduction toward zero, whereas the curve with α= 0.05 μm${}^{-1}$ and $C_{0}=5$ fF shows a monotonical increase. Note that α= 0.2 μm${}^{-1}$, and 0.05 μm${}^{-1}$ correspond to the cases with effective light emission wavelengths of 700 and 850 nm, respectively. In contrast to afterpulsing, crosstalk probability does not necessarily show monotinic dependence on $L_{p}$; the impact of pixel miniaturization is highly dependent on the combination of model parameters.

To suppress crosstalk, several countermeasures can be considered. First, lowering $V_{ex}$ helps to suppress the crosstalk probability at the expense of the PDP and PDE. $V_{ex}$ affects both $N_{ava}$ in the emitter and the sensitivity of the receiver, and hence the crosstalk probability follows the square law with respect to $V_{ex}$. Second, the formation of opaque deep trench isolation (DTI) could suppress the crosstalk. Trench materials with a lower refractive index can reflect the emitted photons and eventually confine the photons in the emitter. This could lead to an order of magnitude improvement of the crosstalk probability.

#### 2.2.6. Power Consumption

**Figure 9:**
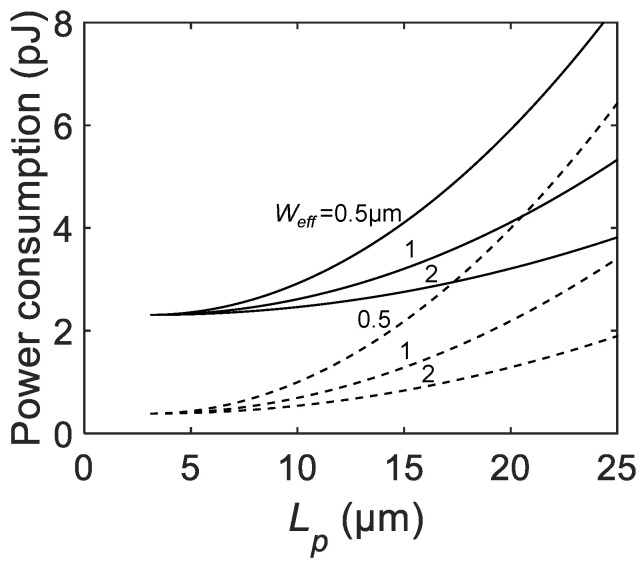
The calculated power consumption as a function of the SPAD pixel pitch $L_{p}$ for $C_{0}=$ 5 fF (dashed lines) and 30 fF (solid lines), active-to-active distance $L_{a-a}=$ 3 μm, $V_{B}=$ 20 V, $V_{ex}=$ 3.3 V, and $W_{eff}=$ 0.5, 1, and 2 μm.

The avalanche-originated power consumption in large-scale SPAD arrays is a key parameter as it grows proportionally to the number of pixels. The total power consumption in a SPAD array depends on the incident photon flux. For a systematic comparison, the following discussion focuses on the energy consumption per single avalanche event, $E_{ava}$, in a single SPAD pixel. The power consumption at the readout circuits is not taken into account here. $E_{ava}$ is a product of $eN_{ava}$ and $\left(V_{ex}+V_{B}\right)$, expressed as follows:

(14) $$\begin{array}{c} E_{ava}=C_{par}\times V_{ex}\times \left(V_{ex}+V_{B}\right)=D\times \left[\frac{\pi \epsilon {\left(L_{p}-L_{a-a}\right)}^{2}}{4W_{eff}}+C_{0}\right], \end{array}$$

where $D=V_{ex}\times \left(V_{ex}+V_{B}\right)$ is the bias-dependent coefficient and $V_{B}$ is the breakdown voltage of the SPAD. Apart from the details of the coefficient, the equation has the same structure as that of the afterpulsing probability. Naturally, the calculated trend of the single-event power consumption $E_{ava}$ as a function of $L_{p}$ in Figure 9 shows similarity to Figure 7.

#### 2.2.7. Timing Jitter

The timing jitter in the SPAD is determined by multiple factors, such as the device configuration, doping profile, detection threshold, excess bias, and temperature, and it is not straightforward to formulate the scaling law for this. Qualitatively, a larger pixel pitch produces a higher timing jitter for several reasons: first, the spatial expansion of the avalanche multiplication process takes more time in the larger $L_{p}$ due to the finite lateral avalanche propagation velocity [39]. Second, a larger $L_{p}$ requires slower rising of the output voltage due to the larger parasitic capacitance, leading to enhanced statistical variability. Further systematic analysis should be conducted for deeper understanding of scaling the timing jitter.

#### 2.2.8. Summary of Scaling Law Analysis

In the above sections, the scaling laws of the key SPAD characteristics with pixel dimensions were investigated. Miniaturization of the SPAD pixel improves the DCR, afterpulsing, power consumption, and timing jitter, whereas it has an adverse effect on the fill factor, PDP, and PDE. The equations for the scaling laws are summarized in Table 1. In particular, the degradation of the single-photon sensitivity is inevitable in the conventional SPAD pixel when its pitch becomes smaller than 10 μm. Further technological breakthroughs are required for SPAD pixel miniaturization toward multi-megapixel arrays.

## 3. Application to Experimental Results

### Extraction of Model Parameters

**Figure 10:**
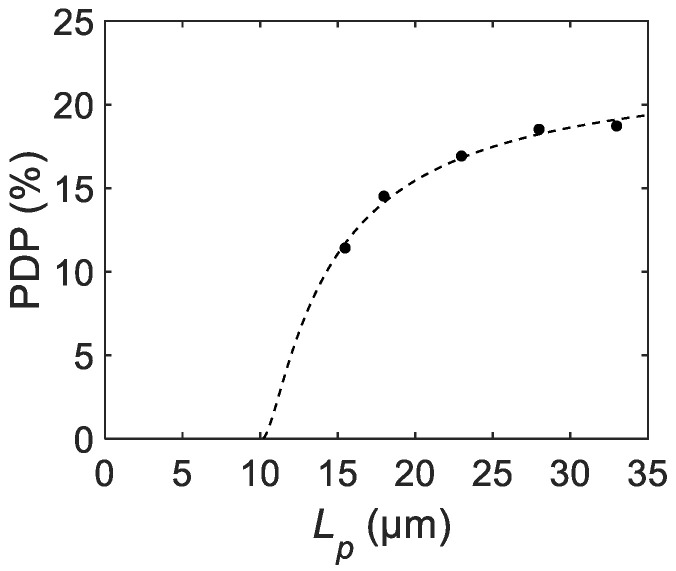
The measured PDP trend from the literature fitted by the theoretical equation [40]. The measured and fitted data are shown as dots and a dashed line, respectively.

To demonstrate the applicability of the scaling law analysis to practical situations, we performed a theoretical fitting with experimental results from the literature. Figure 10 shows experimental data from the literature [40] representing the pixel size dependence of the maximum PDP (shown as dots). Here, $L_{a-a}$ is assumed to be 8 μm. The experimental data was fitted using the scaling law equation for PDP (shown as a dashed line). The fitted curve shows a good agreement with the measurement when the fitting parameters are $PDP_{max}$ = 22.8% and $r_{in}$ = 1.06 μm.

The extracted fitting parameters indicate that the maximum PDP reaches 22.8% for larger pixels with this device configuration and bias condition, whereas the effective photon-insensitive region with the width of 1.06 μm reduces the maximum PDP for smaller pixels. The fitting result implies that the PDP will go down to zero at $L_{p}=L_{a-a}+2\times r_{in}$ = 10.12 μm, and thus the pixel pitch with this SPAD device configuration cannot go below 10 μm unless the process conditions and design rules are modified. Note that $r_{in}$ is determined by the spatial distributions of both the electrostatic potential and photon absorption rate. Given that the latter distribution is a function of the wavelength of incident photons, $r_{in}$ can potentially be dependent on the wavelength of interest.

**Figure 11:**
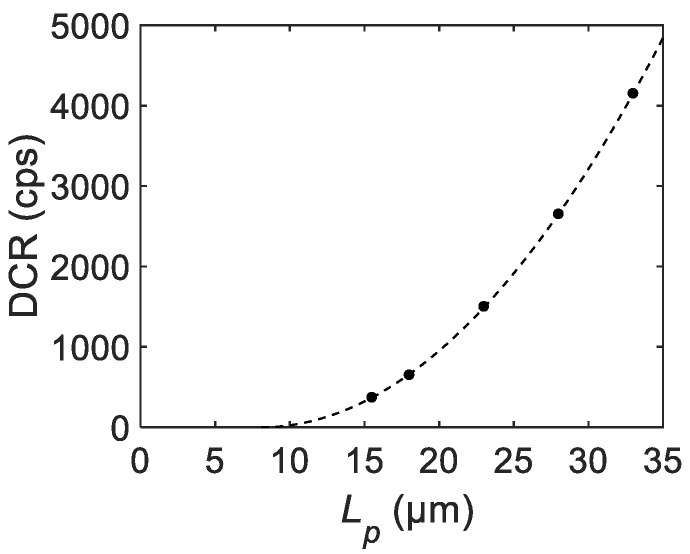
The measured DCR trend from the literature fitted by the theoretical equation [40]. The measured and fitted data are shown as dots and a dashed line, respectively.

In Figure 11, a similar analysis is performed for the measured DCR from the literature. Again, the fitting result shows good agreement with the measurement. The corresponding fitting parameters are extracted as $R_{0}=8.50$ cps/μm${}^{2}$, and $r_{in}=0.030$ μm. An interesting implication is that the extracted $r_{in}$ for the DCR is different from that for the maximum PDP. This can be interpreted similarly to the previous remark on the wavelength dependence of $r_{in}$; the spatial distribution of the photon absorption rate for PDP can be different from that of the thermal generation rate for DCR, thereby, representing a different inactive radius $r_{in}$. The extracted $r_{in}$ for the DCR could provide useful information to estimate the major source of the DCR.

## 4. Discussions

We investigated the theoretical expressions of the scaling laws for the major performance metrics in SPAD pixels. The analysis showed that SPAD pixel miniaturization improved the DCR, afterpulsing, power consumption, and timing jitter, whereas it had an adverse effect on the FF, PDP, and PDE. The scaling law equations for PDP and DCR were then applied to the experimental data in the literature, showing good agreement with the measured trends. The extracted fitting parameters were used to extrapolate the expected pixel size dependence of PDP and DCR, which implied that a pixel size smaller than 10 μm cannot be achieved without modification of the current process conditions and design rules.

Our scaling law analysis has three potential applications: the prediction of SPAD performance based on existing measurement data, extraction of model parameters to quantify the pixel-size-independent metrics, and systematic comparison of SPAD performance tradeoffs for different process, device, and layout configurations. The first approach can be useful for designers to understand the underlying tradeoffs and decide the optimal pixel size for applications of interest. The second approach can be critical to understanding the limiting factors of SPAD performance.

In-depth study of the extracted model parameters provides rich information of SPAD pixels, such as the inactive radius, parasitic capacitance, effective depletion width, and effective decay length of the avalanche-induced photons, which cannot be directly measured with existing measurement techniques. The third approach can be employed for clarifying the pros and cons of one SPAD device configuration to the other, which is essential for the correct choice of process conditions and device structure. Combining these approaches will provide a promising tool for further pushing the limit of SPAD pixel miniaturization toward sub-2 μm-pitch SPADs.

The extracted models are focused on pixel size dependence. Further generalization of the models to fully account for the voltage and temperature dependence of the metrics remains to be verified in future work.

## Figures and Tables

**Table 1:** Summary of the scaling laws in the SPAD pixels with the pixel pitch $L_{p}$ as a scaling parameter. The coefficient is omitted in the equations.

Characteristics	Equation
Fill factor (%)	$\frac{{\left(L_{p}-L_{a-a}\right)}^{2}}{L_{p}^{2}}$
PDP (%)	${\left(\frac{L_{p}-L_{a-a}-2r_{in}}{L_{p}-L_{a-a}}\right)}^{2}$
PDE (%)	$\frac{{\left(L_{p}-L_{a-a}-2r_{in}\right)}^{2}}{L_{p}^{2}}$
DCR (cps)	${\left(L_{p}-L_{a-a}-2r_{in}\right)}^{2}$
DCR density (cps/μm${}^{2}$)	$\frac{{\left(L_{p}-L_{a-a}-2r_{in}\right)}^{2}}{{\left(L_{p}-L_{a-a}\right)}^{2}}$
Afterpulsing probability (%)	$\left[\frac{\pi \epsilon {\left(L_{p}-L_{a-a}\right)}^{2}}{4W_{eff}}+C_{0}\right],$
Crosstalk probability (%)	$\left[\frac{\pi \epsilon {\left(L_{p}-L_{a-a}\right)}^{2}}{4W_{eff}}+C_{0}\right]\times \frac{e^{-\alpha L_{p}}}{L_{p}^{2}}\times \frac{\pi }{4}\times {\left(L_{p}-L_{a-a}-2r_{in}\right)}^{2}$
Power consumption (pJ)	$\left[\frac{\pi \epsilon {\left(L_{p}-L_{a-a}\right)}^{2}}{4W_{eff}}+C_{0}\right]$

## Data Availability

Not applicable.

## Abbreviations

The following abbreviations are used in this manuscript:

CMOS	Complementary-metal-oxide-semiconductor
DCR	Dark count rate
DTI	Deep trench isolation
FF	Fill factor
PDE	Photon detection efficiency
PDP	Photon detection probability
QE	Quantum efficiency
SPAD	Single-photon avalanche diode
